# Associations between physical activity and quality of life outcomes in adults with severe obesity: a cross-sectional study prior to the beginning of a lifestyle intervention

**DOI:** 10.1186/1477-7525-11-187

**Published:** 2013-11-05

**Authors:** Randi Jepsen, Eivind Aadland, John R Andersen, Gerd K Natvig

**Affiliations:** 1 Faculty of Health Studies, Sogn og Fjordane University College, N-6803 Førde, Norway; 2 Department of Global Public Health and Primary Care, University of Bergen, N-5020 Bergen, Norway; 3 Førde Health Enterprise, N-6807 Førde, Norway

**Keywords:** Severe obesity, BMI, Quality of life, Life satisfaction, SF-36, Physical activity, Accelerometer

## Abstract

**Background:**

Severely obese individuals who seek lifestyle interventions have impaired quality of life (QoL). Research suggests that physical activity (PA) plays a role in weight reduction and improved health in this group, but knowledge about the association of PA with QoL outcomes is sparse and inconsistent. The aim of this study was to investigate whether a higher level of PA was independently associated with higher QoL in severely obese individuals prior to the beginning of a lifestyle intervention.

**Methods:**

During 2010, a total of 49 severely obese individuals who began a lifestyle intervention programme in Western Norway agreed to participate in the study. Data were collected prior to the beginning of the intervention. QoL was measured by a one-item scale on life satisfaction and the SF-36, PA was measured by an accelerometer, and clinical data were collected by health staff. Linear regression analyses were used to determine the associations between PA and QoL outcomes (life satisfaction, physical functioning, and mental health), adjusting for age, gender, and body mass index (BMI).

**Results:**

In the adjusted analyses, we found positive relationships between PA and life satisfaction (Stand. coeff. 0.39, *p* = 0.024) and physical functioning (Stand. coeff. 0.34, *p* = 0.025). There was no association between PA and mental health (Stand. coeff. 0.15, *p* = 0.376).

**Conclusion:**

This study detected associations between objectively measured PA and life satisfaction as well as physical functioning in a group of severely obese individuals before they began a lifestyle intervention programme.

## Introduction

Severe obesity affects a growing proportion of individuals [1]. Its direct cause is an imbalance in intake (diet) and expenditure (physical activity (PA)) of energy, but the underlying mechanisms include complex political, environmental, sociocultural, genetic, and personal factors. Medical consequences, such as type 2 diabetes, cardiovascular disease, and certain types of cancer, contribute to morbidity and mortality in the severely obese population [2]. Moreover, psychosocial problems are common [3], and, although severely obese individuals do not constitute a homogenous group, many report poorer quality of life (QoL) compared to the general population [4,5]. QoL is a global, multidimensional construct representing overall relative satisfaction with life. In health research, life satisfaction is measured either with a single item or with multi-item scales. Health-related quality of life (HRQoL) is limited to QoL aspects that are related to health and illness [6,7]. It is often assessed with questions that are relevant across populations and conditions [8].

Severely obese individuals with a desire to ameliorate their situation may seek lifestyle interventions. This desire seems to be related to impaired QoL, growing health problems, and reduced functioning [4,5]. Seeking a lifestyle intervention should imply a decision about increasing one’s level of PA, which usually constitutes one of the components of this type of programme [9]. PA promotes and helps to maintain weight reduction in obese individuals, although the strength of the effect is debated [10,11]. In addition, PA protects against medical conditions such as cardiovascular disease and type 2 diabetes in all Body Mass Index (BMI) categories [12,13]. Nevertheless, despite the potential benefits, it seems very difficult for obese individuals to increase their PA levels, especially in the long run [14]. Improved health has a long-term perspective [12,13]. Weight loss is a less distant outcome, but expectations are often unrealistically high and may result in disappointment [15,16]. As a motivational factor, it may be more useful to maintain a realistic short-time effect, and improved QoL may play a role in this regard. Thus, it is vital to determine whether PA is associated with QoL outcomes in severely obese individuals. Such a relationship has been detected for the physical domain in individuals awaiting gastric-bypass surgery [17] or other obesity treatment [18] and in the mental and physical domains in overweight to obese subjects [19]. Wang et al. found associations between PA and the physical domain in a study of overweight and obese adults at the beginning of a behavioural weight loss trial [20]. However, to our knowledge, no studies have targeted severely obese subjects seeking lifestyle interventions. Therefore, the aim of this study was to investigate the associations among objectively measured PA and life satisfaction, physical functioning, and mental health in severely obese subjects prior to the beginning of a lifestyle intervention programme.

## Methods

### Design and participants

This study is part of an observational cohort study with a two-year follow-up and a variety of data. In the present study, we included baseline data for 49 adults with severe obesity who began a two-year lifestyle intervention at The Red Cross Haugland Rehabilitation Centre (RCHRC) in Western Norway during 2010. After inclusion of the 49 participants, the intervention was radically changed, which hampered further inclusion.

Inclusion criteria were age 18–60 years and BMI ≥ 40 kg/m^2^ with or without comorbidities or ≥ 35 kg/m^2^ with comorbidities at the time of referral (i.e., individuals included in the right to treatment in the Norwegian public specialist health service). Exclusion criteria were referral to or previous obesity surgery, pregnancy, severe cardiovascular disease, alcohol or substance abuse, or mental illness or physical impairment that prevented the participants from adhering to the intervention. All of the patients accepted for the intervention were eligible for inclusion in the study.

### Setting

The intervention included four residential periods and was managed by a multi-professional team. It combined PA, a balanced diet, and cognitive behavioural therapy. The desired outcomes were improved QoL, better general health, weight loss, and reduction of obesity-related medical problems.

### Ethical approval

Verbal and written information about the study was provided by the staff at the rehabilitation centre, and written informed consent was obtained from each participant prior to the study. This study met the standards of the Declaration of Helsinki and was approved by the Regional Committee for Medical and Health Research Ethics (registration number 2010/159a).

### Measures and procedures

#### Data collection

All data were collected prior to the beginning of the lifestyle intervention. PA was measured four weeks prior to the first residential stay in the rehabilitation centre, and all other data were collected on arrival. Anthropometric data were obtained by trained health staff, and all questionnaires were self-administered.

#### Quality of life outcomes

Life satisfaction was measured with a single item on a seven-step scale with alternatives from “very satisfied” to “very dissatisfied”. The scores were reversed before data analysis so that higher scores indicated higher life satisfaction. The scale on life satisfaction has been widely applied in Norwegian population studies. It has shown predictive value for later onset of type 2 diabetes [21] and strong direct associations with poor self-reported health, mental problems, and lack of social relations [22]. HRQoL was measured with the multidimensional Medical Outcomes Study Short-Form 36 Health Survey (SF-36) version 1.2. From its 36 items, eight sub-domains were derived in accordance with the standard procedure for SF-36 subscale scoring. Each scale ranged from 0 (worst) to 100 (best) [8]. The SF-36 has demonstrated good reliability and validity in obesity research [4]. The subscale on physical functioning has ten items related to self-care, mobility, and light and strenuous activities, whereas the mental health subscale comprises five questions about positive as well as negative emotions and mood states [8]. The other six subscales of the SF-36 (role physical, bodily pain, general health, vitality, social functioning, and role emotional) served as secondary outcomes.

#### Socio-demographic information

The participants gave information on age, gender, civil status (“married/cohabiting” versus “single/divorced”), educational level (“< 15 years” versus “≥ 15 years of schooling” (i.e., college/university)), and employment (“employed” versus “not working” (i.e., being unemployed or receiving pensions or benefits)).

#### Anthropometry

Body weight and fat mass were measured on a bioelectrical impedance analysis device (BC 420S MA, Tanita Corp, Tokyo, Japan) in the morning, in light clothes, in a fasting state, and after voiding. Weight was reported to the nearest 0.1 kg. Height was measured without shoes to the nearest 0.5 cm using a stadiometer. Waist circumference was measured twice at the level of the umbilicus at exhalation and reported as the mean value of the two measurements. BMI was calculated as weight in kilograms divided by the square of height in meters.

#### Physical activity

Free-living PA was measured with the Actigraph GT1 M accelerometer (Actigraph, Fort Walton Beach, FL, USA). This accelerometer is a frequently used hip-worn electronic motion sensor. Acceleration is converted into activity counts that increase linearly with the magnitude of the acceleration and work rate. The participants were instructed to wear the accelerometers for seven full days, except during water activities or while sleeping. A wear-time of ≥ 10 hours/day for ≥ four days was used as the criterion for a valid measure. Non-wear time was defined as periods of ≥ 60 consecutive minutes with zero counts, with allowance for two minutes of counts greater than zero. Data were analysed with the Actigraph software ActiLife v. 5.3. The counts were summed and averaged over the total wearing time to indicate the overall PA and reported as total counts per minute. Accelerometer-measured PA is considered to have superior validity compared to self-reported information [23].

### Statistical analysis

The data were analysed using SPSS for Windows (Version 20.0. Armonk, NY: IBM Corp). Categorical variables are presented as percentages, and continuous variables are presented as means and standard deviations (SD). For unadjusted and adjusted linear regression, only participants with complete data sets were included. Three separate unadjusted and adjusted linear regression analyses were performed to evaluate the associations between PA and QoL outcomes. Gender, age, and BMI served as covariates in the adjusted analyses. A two-sided p-value < 0.05 indicated statistical significance.

## Results

### Sample characteristics

Forty-nine (92.5%) of the 53 invited patients agreed to participate in the study. The data collection was complete for all 49, except from accelerometer-measured PA, for which valid data were obtained from 42 participants. In addition, there was one missing measure of waist circumference. The missing data on PA were due to two cases of invalid measurements and five with no measurements at all. Socio-demographic characteristics, anthropometrics, PA, and scores on QoL outcomes are presented in Table 1.

**Table 1:** Characteristics of the participating adults with severe obesity

Age, mean (SD), *N* = 49			43.6 (9.4)
Gender, n (%), *N* = 49			
	Women		37 (75.5)
Socio-demographic status, n (%), *N* = 49			
	Married/cohabiting		30 (61.2)
	Having children		27 (55.1)
	Formal education ≥ 15 years		22 (44.9)
	Employed		41 (83.7)
Anthropometrics, mean (SD)			
	Body mass index, kg/m^2^, *N* = 49		42.1 (6.0)
	Fat mass, percent, *N* = 49		47.0 (6.2)
	Waist circumference, cm, *N* = 48		128.3 (13.0)
Physical activity, mean (SD), *N* = 42			
	Accelerometer-measured counts per minute		280 (100)
Quality of life outcomes, mean (SD), *N* = 49			
	Main outcomes		
		Life satisfaction^a^	4.6 (0.9)
		Physical functioning^b^	72.1 (21.0)
		Mental health^b^	73.7 (13.7)
	Secondary outcomes		
		Role physical^b^	65.8 (38.1)
		Bodily pain^b^	62.5 (25.8)
		General health^b^	61.0 (20.7)
		Vitality^b^	45.0 (20.7)
		Social functioning^b^	79.8 (22.4)
		Role emotional^b^	73.5 (36.6)

^a^Life satisfaction (scale 1–7; higher scores represent higher life satisfaction).

^b^Medical Outcomes Study Short-Form 36 Health Survey (scale 0–100; higher scores represent better quality of life outcomes).

### Main and secondary outcomes

Increased PA levels were associated with higher life satisfaction and physical functioning in both unadjusted and adjusted models (Table 2). In the adjusted analyses PA had a stronger association with life satisfaction (Stand. coeff. 0.39, *p* = 0.024) than with physical functioning (Stand. coeff. 0.34, *p* = 0.025). According to Cohen 0.3 is the cut-off point for a medium effect size, which applies to both associations [24]. In contrast, there was no association between PA and mental health. Lower BMI was related to better physical functioning in the unadjusted analysis, but not the adjusted analysis. Male gender predicted better physical functioning in both analyses. Age had no associations with any of the QoL outcomes. Of the six subscales of the SF-36 defined as secondary outcomes, only general health showed a statistically significant association with PA (unadjusted: Stand. coeff. 0.31, *p* = 0.045; adjusted: Stand. coeff. 0.35, *p* = 0.042).

**Table 2:** **Regression coefficients (Reg. coeff.) with 95% confidence interval (CI) and standardised coefficients (Stand. coeff.) for unadjusted and adjusted**^**a**^**linear association of quality of life outcomes in severely obese adults**

		**Life satisfaction**^**b**^			**Physical functioning**^**c**^			**Mental health**^**c**^		
		***N*** **= 42**			***N*** **= 42**			***N*** **= 42**		
		**Reg. coeff. (95% CI)**	**Stand. coeff.**	***p***	**Reg. coeff. (95% CI)**	**Stand. coeff.**	***p***	**Reg. coeff. (95% CI)**	**Stand. coeff.**	***p***
Gender = male										
	Unadjusted	0.31 (−0.29, 0.90)	0.16	.306	14.8 (0.9, 28.6)	0.32	**.037**	−1.5 (−12.1, 9.0)	−0.05	.771
	Adjusted	0.45 (−0.15, 1.05)	0.24	.140	15.2 (2.5, 27.8)	0.33	**.020**	1.3 (−9.6, 12.1)	0.04	.815
Age										
	Unadjusted	−0.00 (−0.03, 0.03)	0.01	.928	−0.2 (−0.09, 0.4)	−0.11	.501	0.4 (−0.1, 0.8)	0.24	.122
	Adjusted	0.01 (−0.02, 0.03)	0.09	.599	−0.2 (−0.7, 0.4)	−0.08	.580	0.5 (−0.4, 0.9)	0.24	.069
Body mass index										
	Unadjusted	−0.00 (−0.05, 0.04)	−0.03	.844	−1.3 (−2.2, -0.3)	−0.40	**.008**	0.3 (−0.5, 1.0)	0.12	.454
	Adjusted	0.02 (−0.03, 0.06)	0.13	.446	−0.9 (−1.8, 0.04)	−0.28	.060	0.6 (−0.3, 1.3)	0.23	.191
Physical activity^d^										
	Unadjusted	0.003 (0.000, 0.005)	0.31	**.047**	0.08 (0.02, 0.13)	0.39	**.011**	0.01 (−0.04, 0.05)	0.04	.787
	Adjusted	0.003 (0.000, 0.006)	0.39	**.024**	0.07 (0.01, 0.12)	0.34	**.025**	0.02 (−0.03, 0.07)	0.15	.376
Adjusted R^2^		0.06			0.29			0.01		

Significant p-values (< 0.05) in bold.

^a^All variables in the first column.

^b^Continuous scale. Higher scores represent higher life satisfaction.

^c^Medical Outcomes Study Short Form 36 Health Survey subscales. Continuous scales. Higher scores represent higher health-related quality of life.

^d^Accelerometer measured.

## Discussion

In this cross-sectional study of associations between PA and QoL outcomes in severely obese adults prior to the beginning of a lifestyle intervention, the main findings were that PA was positively associated with life satisfaction and physical functioning independent of age, gender, and BMI, but it was not associated with mental health. The association was stronger for life satisfaction than for physical functioning.

Previous studies have shown a similar association between PA and physical functioning [17-20]. However, none of these studies are directly comparable to the present study. First, the previous studies used self-reported data on PA, not objectively measured data. Second, the participants may differ considerably. Lerdal et al. studied severely obese waiting-list patients who were not yet referred for either obesity surgery or lifestyle intervention [18], whereas Bond et al. reported on obesity surgery candidates [17]. Wang et al. recruited overweight and obese adults for a behavioural intervention trial, so their subjects’ BMI was considerably lower compared to the present study [20]. Martin et al. studied the effect of exercise sessions rather than all-day activities on overweight to obese sedentary women [19]. Lastly, a review by Bize et al. of population studies concluded that there seemed to be an association between PA and the physical domain of HRQoL, but the authors did not consider BMI, and PA was self-reported in most of the included original studies [25]. Nevertheless, from a conceptual perspective, it seems plausible that a scale ranging from light daily functioning to more strenuous activities, such as climbing several stairs and running, is related to PA in severely obese individuals [8].

It was interesting that PA showed the strongest positive association with life satisfaction amongst the QoL outcomes we used. Compared to limitations in physical functioning, which are concrete and related to daily activities, life satisfaction is a relative concept that includes all major dimensions of life. As opposed to a negative approach concerned with problems, limitations, and suffering, the subjective assessment of life satisfaction takes a positive point of departure [6,7]. In the present study, life satisfaction was represented by a single question. Similarly, Idler and Benyamini found that self-rated health, measured by one question, predicted mortality in almost all of the 27 community studies they reviewed, indicating that single-item scales possess the power to capture the complexity of global concepts [26].

The global character of life satisfaction also differs considerably from the narrow SF-36 mental health scale, with its five items related to current emotions such as anxiousness, depressive moods, and calmness [8]. Therefore, it should not be assumed that life satisfaction shares associations with mental health. Moreover, our finding that PA was not associated with mental health is in line with the study by Lerdal et al. on severely obese waiting-list patients [18], whereas Martin et al. found such a relation in their sample of overweight to obese sedentary women [19]. In the original studies reviewed by Bize et al. the associations between PA and mental health were inconsistent between studies, and, as mentioned, the authors did not include BMI as a variable in the review [25].

Distress works as an incitement for care-seeking [7]. Thus, studies have revealed that severely obese individuals seeking lifestyle interventions experience impaired QoL, health problems, and reduced functioning. Although this distress is not to the same degree as that of obesity surgery seekers, it is still significantly more than the group of severely obese people who do not seek treatment [4,27]. Our study shows that variation in QoL outcomes within a group of lifestyle intervention seekers is associated with their level of PA. A possible explanation for our finding may be that the participants are situated around a balance point with impairment and the threat of further deterioration on the one side and health-promoting options on the other side. Considering the cross-sectional design of the study, we can only speculate that a higher level of PA contributes to keeping participants on the positive side of this balance point (i.e., contributes to higher life satisfaction and better physical functioning). However, a bidirectional effect may be the case, in which QoL outcomes predict PA level.

Our study may provide the first indication that QoL outcomes could be used as short-term goals related to PA in severely obese individuals in lifestyle intervention programmes. However, intervention studies are needed to determine whether this is true.

### Methodological considerations

By using accelerometers to objectively measure PA, our study is original and has greater validity than previous studies. To the best of our knowledge, we are the first to report such results. Although accelerometers underestimate some activities, they capture walking very well [28], and walking was the most frequent mode of PA in this severely obese population [29].

A second strength of this study is that we used validated instruments to assess QoL outcomes. Instead of using the SF-36 subscales on physical functioning and mental health, we could have chosen the physical and mental summary scores, which summarise more items into two broader components [30]. A drawback of the summary scores is that they are less distinct than the subscales; therefore, we suspect that they may be less valid for the purpose [8]. However, Bond et al. found associations between PA and the physical summary score in their study of gastric-bypass candidates [17]. This should be examined in future research.

This study has several limitations. First, due to the limited size of the cohort in the intervention programme, the number of participants was quite small restricting the number of covariates included in the regression analyses. Furthermore, the sample was gender biased in that there were few men. Consequently, we could not determine if there were gender differences in the associations between PA and QoL outcomes. This issue should be examined in larger samples. However, the gender bias in the current study is typical in the obese population seeking lifestyle interventions [4]. Additionally, although the inclusion rate was high (92.5%), not all participants were included in the regression analysis due to missing accelerometer data. Wearing an accelerometer for seven days requires effort, and there may be many reasons for not strictly adhering to the procedure.

As mentioned, because our study was cross-sectional, we cannot assume any causal relationships among the variables. Moreover, we do not know whether an increase in PA will improve QoL outcomes in the long term. Longitudinal designs and intervention studies should be conducted to examine these issues.

The participants in this study are most likely not representative for all severely obese individuals. As mentioned, lifestyle intervention seekers are known to differ from obesity surgery and non-treatment seekers, so the results cannot be generalised to the severely obese population as a whole [4,27].

## Conclusion

In this cross-sectional study of severely obese adults prior to the beginning of a lifestyle intervention, we found positive associations between PA and life satisfaction as well as physical functioning. The association was stronger for life satisfaction than for physical functioning. Further research is needed to determine causal relationships between the variables, and intervention studies should be performed to evaluate the possible effect of PA on QoL in severely obese individuals in lifestyle intervention programmes.

## Abbreviations

BMI: Body mass index; HRQoL: Health-related quality of life; QoL: Quality of life; RCHRC: Red Cross Haugland rehabilitation centre; SPSS: Statistical package for the social sciences; SF-36: Medical outcomes study short-form 36 health survey.

## Competing interests

The authors declare that they have no competing interests.

## Authors’ contributions

RJ participated in the design of the study, analysed the data, and drafted the manuscript. EA participated in the design of the study, collected data, analysed the data, and revised and helped draft the manuscript. JRA participated in the design of the study, analysed the data, and revised and helped draft the manuscript. GKN participated in the design of the study, helped with the statistical analysis, and revised and helped draft the manuscript. All authors read and approved the final manuscript.
